# Limb-girdle muscular dystrophy 1F is caused by a microdeletion in the transportin 3 gene

**DOI:** 10.1186/1750-1172-10-S2-O20

**Published:** 2015-11-11

**Authors:** Saida Ortolano

**Affiliations:** 1 Group of Neonatal and Pediatric Pathology/Rare Diseases, Instituto de Investigación Biomédica de Ourense-Pontevedra-Vigo (IBI) Hospital Rebullón, Vigo Spain

## 

Whole genome sequencing strategy allowed identifying the gene responsible for autosomal dominant limb-girdle muscular dystrophy 1F, which was previously linked to locus 7q32.1-32.2. A large Spanish family spanning six generations with limb girdle muscular weakness and distal involvement was find to present a mutation in the stop codon of *TNPO3* gene (c.2771delA). The mutation segregates with the clinical phenotype, and is absent in healthy relatives of the family as well as in genomic sequence databases. Histological abnormalities of the nuclei and altered *TNPO3* expression assessed in muscle biopsy of the patients indicate impaired *TNPO3* function. *TNPO3* encodes transportin-3, a serine/arginine rich protein carrier through nuclear membrane. The function of transportin-3 in skeletal muscle has not been thoroughly characterized. The identification of this mutation as the cause of autosomal dominant limb-girdle muscular dystrophy highlights the importance of defects of nuclear envelope proteins as causes of inherited myopathies[1].
